# Protein corona – from molecular adsorption to physiological complexity

**DOI:** 10.3762/bjnano.6.88

**Published:** 2015-03-30

**Authors:** Lennart Treuel, Dominic Docter, Michael Maskos, Roland H Stauber

**Affiliations:** 1 Fraunhofer ICT-IMM, Carl-Zeiss-Str. 18-20, 55219 Mainz, Germany; 2 Physical Chemistry, University of Duisburg-Essen, Universitaetsstr. 5–7, 45117 Essen, Germany; 3 Molecular and Cellular Oncology/Mainz Screening Center (MSC), University Hospital of Mainz, Langenbeckstrasse 1, 55101 Mainz, Germany

**Keywords:** agglomeration, corona composition, dynamics, nanoparticles, protein corona

## Abstract

In biological environments, nanoparticles are enshrouded by a layer of biomolecules, predominantly proteins, mediating its subsequent interactions with cells. Detecting this protein corona, understanding its formation with regards to nanoparticle (NP) and protein properties, and elucidating its biological implications were central aims of bio-related nano-research throughout the past years. Here, we discuss the mechanistic parameters that are involved in the protein corona formation and the consequences of this corona formation for both, the particle, and the protein. We review consequences of corona formation for colloidal stability and discuss the role of functional groups and NP surface functionalities in shaping NP–protein interactions. We also elaborate the recent advances demonstrating the strong involvement of Coulomb-type interactions between NPs and charged patches on the protein surface. Moreover, we discuss novel aspects related to the complexity of the protein corona forming under physiological conditions in full serum. Specifically, we address the relation between particle size and corona composition and the latest findings that help to shed light on temporal evolution of the full serum corona for the first time. Finally, we discuss the most recent advances regarding the molecular-scale mechanistic role of the protein corona in cellular uptake of NPs.

## Introduction

In biological environments, proteins adsorb to the surfaces of nanoparticles (NPs), thus, forming a protein layer around the particles: the protein corona. Subsequent interactions between the NP and biological entities are mediated by the presence and nature of this corona. The relation between the original surface functionality of the NP and the nature of the corona is far from trivial and still remains very elusive [1–7]. It has been shown that not only the NP surface chemistry but also features such as NP size [8] and charge [4,9] are critical factors determining the formation and nature of the protein corona.

The composition and molecular properties of the protein corona have been shown to be influential factors for the cellular uptake of NPs [7,10–12] but direct links between structure and effect remain to be established. In the following paragraphs, we will briefly discuss the parameters that were so far identified to affect NP–protein interactions and evaluate their possible implications. Then, we will highlight specific, detailed, aspects that were in the focus of our own experimental work within the SPP1313.

Focusing on the NP, ligand exchange under physiological conditions is an important point. Many ligands can easily be replaced on NP surfaces in equilibrium exchange-reactions [13]. Such approaches are frequently utilized in NP synthesis, in which specific ligands are often needed to synthesize NPs with desired properties, but are subsequently replaced by other ligands, e.g., to alter the solubility or avoid adverse effects related to toxic ligands [13–16]. Protein adsorption can well lead to the exchange of small monomer-type ligands on NP surfaces under physiological conditions while other surface functionalizations, such as polymer coatings, may be persistently wrapped around the NP and cannot be exchanged. We point out that this leads to a situation in which not only the chemical nature of the ligand but also its persistence on the NP surface can be decisive for the biological fate of the NP.

The effect of changing surface energy or surface restructuring is well-known from the field of catalysis [17–21] but its implications for the biological behavior of NPs remains somewhat elusive. We note that the macromolecular nature of the proteins constituting the corona requires a broader view of the situation: While single atoms or atomic scale surface defects are of high importance for the adsorption of small molecules that are frequently considered in catalytic mechanisms, the size of the adsorbing protein, typically several nanometers, leads to an interaction process averaging over many different atomic surface sites, hence the individual influence of single surface sites is reduced. The extent to which this effect governs the overall behavior observed for protein adsorption to NP surfaces remains unknown, largely due to elusive characteristics of the NP surface under physiological conditions on an atomic scale. Hydrophilic and/or hydrophobic properties of NPs are also difficult to assess on an atomic scale under the chemically complex conditions of physiological media. These properties can also be critically affected by protein adsorption, leading to a situation in which the NPs would, mediated by the protein corona, reach biological “endpoints” that they could not reach without their protein cover [5,7,22].

Dissolution of NPs has also been addressed and is of specific importance where molecular or ionic substances are released from the NP that cause own, sometimes well known, adverse effects [23–27]. The intriguing consequence of dissolution is that the particulate state may define the transport of the NPs within a biological system and molecular agents that are released wherever the NPs are located may dominate the (patho)biological effects. In consequence, the delicate interplay between the relative timescales of particle transport and dissolution/release kinetics can well govern NP toxicity. While this factor further complicates a fundamental understanding of NP toxicity, the right time-scale ratio of the participating effects can be a critically important parameter for the design and synthesis of nano-medical applications [28].

Some NPs (e.g., Au, Pd, Pt) are known as good catalysts in a range of different reactions [14,17,21,29–35]. A relevant example in the context of protein adsorption is the catalytic effect of Au NP surfaces on the formation/cleavage of disulfide (S–S) bridges that can also occur in corona proteins [36–37]. The mechanisms of this catalysis, especially under physiological conditions, remain elusive but electron–hole pairs have been suggested to contribute to redox processes on NP surfaces [1,26,38]. A recent theoretical study even suggests that disulfide bonds are affected by redox reactions without electron transfer [37]. A further prominent example of NP-associated catalytic activity are iron-catalyzed reactions such as the Fenton reaction [39] and others [10–11] that have been widely investigated [34,40]. Such effects are important in two different aspects: They may change adsorbed proteins but also NPs may act as local catalysts in biological reactions. The manifold of potential consequences resulting from such catalytic action and the relevance to the biological system are difficult to predict. More detailed investigations under specific conditions of biological systems are needed to allow for a more educated assessment.

A further very important aspect of corona formation is its impact on colloidal stability: Under physiological conditions, many NPs lose their original stabilization due to high electrolyte concentrations and sometimes even due to interaction with biomolecules. Not only do the subsequent agglomeration processes lead to a loss of accessible surface area, they also lead to changes in diffusion properties and, in case of larger agglomerates, give rise to sedimentation [41]. This becomes a critical problem in many in vitro studies, in which the actual dose rate to the cells may be strongly affected by agglomeration kinetics and subsequent sedimentation rates rather than the original NP concentration [41].

Particle size and surface curvature have also been identified as an influential factors shaping the protein adsorption to NP surfaces [42–46] and it has been demonstrated by Tenzer, Stauber and co-workers [8], how NP size, as a single factor, can determine the composition of the physiological protein corona.

Structural changes of the protein may occur upon adsorption onto NP surfaces resulting in altered protein conformations [6,47–59]. Such altered conformations may well lead to the exposure of cryptic peptide epitopes [60–61], altered function and/or avidity effects [22,43,62–64]. When cryptic epitopes are exposed as a consequence of proteins being denatured on a particle surface, an immune response [65–66] may result, which could promote autoimmune diseases if directed against a self-protein [1]. Also related to structural changes, fibrillation of proteins has been reported under specific conditions [67–68] and the possible effect of NPs on protein fibrillation was also discussed [35,62].

Another likely consequence of structural changes in the protein upon adsorption is an altered solubility [69–70]. As the structures of most serum proteins are optimized to combine optimal function and good solubility, changes in this structure can result in a drastically changed solubility. The exact qualitative and, more so, quantitative consequences of this effect for the treatment of protein adsorption/desorption as equilibrium remain elusive. The general validity of such equilibrium treatments has recently been convincingly demonstrated by Treuel, Nienhaus and co-workers [4]. It should, however, be pointed out that the equilibrium nature of this process may strongly depend on both, NP, and protein and can significantly change with their individual properties. The thermodynamic and biological driving forces as well as the mechanistic details of protein unfolding at NP surfaces remain still elusive [1,71–73].

To study the structure of proteins in solution and adsorbed onto NP surfaces, a large range of experimental techniques has been employed [74–75]. This includes spectroscopic techniques such as fluorescence spectroscopy [76–77], Fourier transform infrared spectroscopy [78], Raman spectroscopy and surface-enhanced Raman spectroscopy (SERS) [36,79] as well as circular dichroism spectroscopy [6,47,53,80–81]. Also, other established techniques were used to study protein adsorption such as isothermal titration calorimetry (ITC) [82–83], and surface plasmon resonance (SPR) [22,84]. Moreover, techniques based on the size of proteins and protein–NP complexes have been utilized such as size-exclusion chromatography (SEC) [85–90] or one- and two-dimensional polyacrylamide gel electrophoresis (1D-/2D-PAGE) [28,91–98].

Recently, Tenzer et al. [8,99] introduced an intriguing combination of 1D-/2D-PAGE and immune-blotting with sophisticated label-free liquid chromatography mass spectrometry (LC-MS) and demonstrated the unique abilities of this approach to quantify the composition of the protein corona under complex conditions. This technique represents a major contribution to the toolbox for protein corona studies and paves the way for sophisticated and detailed studies under complex physiological conditions.

With this review, we seek to describe the current state of knowledge regarding the corona formation on a molecular level. The link between the very basic physicochemical knowledge that can be obtained in simplified systems and the observations made under physiological conditions needs to be established and we discuss the current achievements and remaining gaps.

## Review

### Colloidal stability and the corona

Many NPs are found to aggregate in media that contain electrolytes in high concentrations [74,100–103]. To prevent NPs from agglomeration upon collision with one another, they are usually stabilized in solution by a repulsive barrier created by electrostatic or steric repulsion [104–105]. While the former depends on NP charge and is destroyed to a large extent by the presence of electrolytes, as well-known from the Derjaguin–Landau–Verwey–Overbeek (DLVO)-theory [106–107], the latter is largely independent of charge. NP agglomeration in presence of more complex molecular species is far from being completely understood [108–109] but relations between colloidal stability and protein corona formation were reported [3,12,22,43,110–112] and attributed to a to steric stabilization [113].

**Figure 1:**
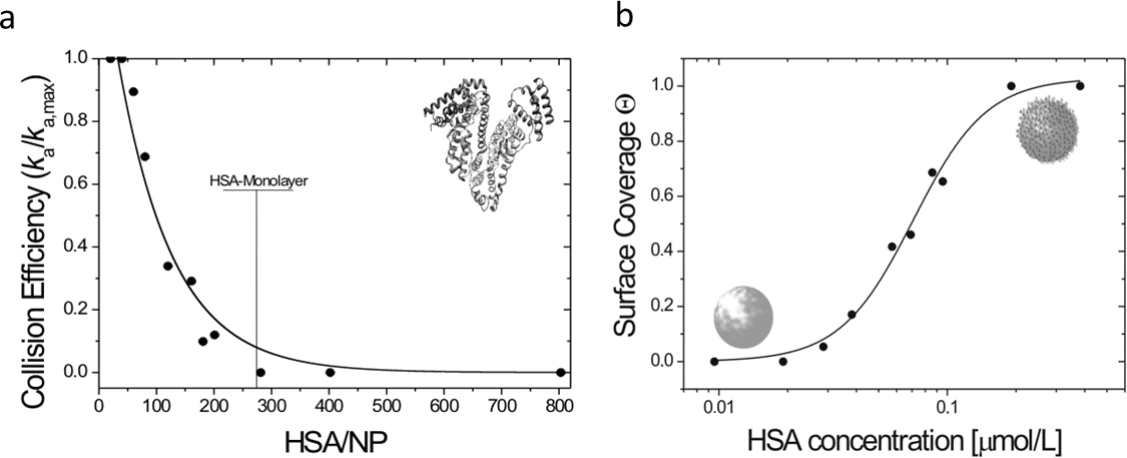
(a): Relation between the collision efficiency regarding the formation of agglomerates in a destabilized colloidal and the relative HSA concentration in solution [53]. Black dots: data points, black solid line drawn to guide the eye. The collision efficiency becomes zero around the point where monolayer surface coverage can be expected. (b): Data-plot of surface coverage versus the logarithmic HSA-concentration from the same study [53]. Surface coverage was inferred from the stabilizing effect of the HSA corona forming around the deliberately destabilized colloid. Black dots: data points, black solid line: Hill fit to the data points (*K*_D_ = 71 ± 17 nmol·L^−1^, *n* = 2.71) indicating a cooperative binding behavior. Reproduced with permission from [53]. Copyright 2012 American Chemical Society.

Recently, Gebauer, Treuel and co-workers [53] presented an in-depth investigation of the quantitative effect of corona formation on colloidal stability. By carefully increasing the ionic strength of the surrounding medium, Gebauer et al. [53] removed the charge stabilization of a citrate-coated Ag colloid, and measured the resulting agglomeration rate. Since the agglomeration process primarily consists of binary collisions between particles, the rate can be described by a simple second-order rate process as derived before [108,114–115]. Testing the effect of corona formation on the agglomeration rate, they revealed a decrease of the collision efficiency (i.e., the overall number of collisions divided by the number of collisions that form an agglomerate) with an increasing protein (human serum albumin, HSA) concentration (Figure 1a). Intriguingly, they found that their colloid was stable at a point where enough proteins were present in solution to cover all NP surfaces in a monolayer fashion.

By introducing a new model, based on statistical considerations of the collision geometries, they showed that a plot of the surface coverage versus the logarithmic protein concentration indeed resembled a binding curve. The subsequent evaluation of this curve with a fitting routine using the Hill equation [111,116–118] (Figure 1b) allowed a quantitative derivation of a binding affinity (*K*_D_ = 71 ± 17 nmol·L^−1^).

Such *K*_D_ values for protein adsorption/desorption onto NP surfaces are extremely useful parameters for a comparable biophysical understanding of NP–protein interactions [4–7,9,12,47,53,81,111]. They should, however, generally be interpreted with caution: While convincing evidence has been presented for the true equilibrium nature of the NP–protein association/dissociation process [4] it remains unclear if this interpretation is fully valid for all NP–protein systems.

A very important aspect resulting from the work by Gebauer et al. [53] is the concept that stability of NPs under physiological conditions can be almost entirely independent of the as-synthesized particle formulation. Here, the authors demonstrated how charge stabilization can be replaced by steric effects providing new and altered stability to the NPs. Such mechanistic insights are critically important for the molecular scale interpretation of the effects resulting from the complex interplay between thousands of individual proteins in the physiological corona. This is, however, not a straight forward task, as many colloids are well known to be unstable in serum [7,53,102,113] suggesting contributions of additional parameters. The factual situation under physiological conditions will likely consist of different protein effects with some proteins exerting a stabilizing influence and others introducing destabilizing properties. Even selective bridging effects between individual proteins could contribute to a possible destabilization.

### Role of individual bonds in NP–protein interactions

As discussed above, little is known about the mechanistic details of protein unfolding upon adsorption onto NP surfaces. Supposedly, different degrees of denaturation and different mechanisms will be involved, depending on the chemical and physical nature of the NP surface and on that of the protein.

Grass and Treuel employed surface enhanced Raman spectroscopy (SERS) to elucidate mechanistic aspects on insulin adsorption onto Au nanoshells [36]. SERS is a very powerful technique to study the adsorption of molecules on metallic nano-surfaces [119–122] and has been described in great detail [123–126]. In the context of protein adsorption, it needs to be pointed out that the enhancement effect in SERS strongly depends on the distance between the Raman/SERS active bond and the surface of the SERS substrate [121,123–127].

**Figure 2:**
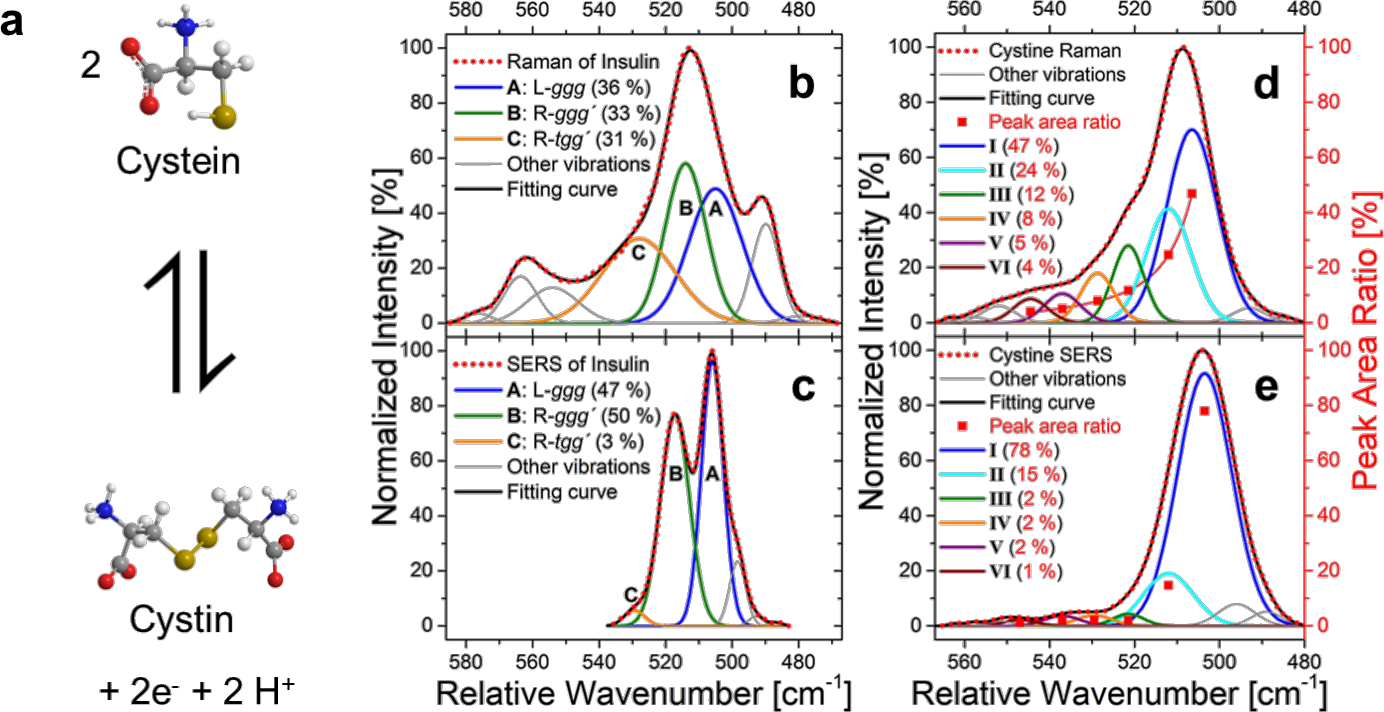
(a): Schematic representation of the dimerization redox equilibrium between cystein and cystin. (b) Normalized Raman and (c) SERS spectra of insulin deconvoluted by multiple Gaussians (disulfide stretching mode region). Relative experimental ratios of the Gaussian areas corresponding to the different S–S bond rotamers are indicated in brackets. (d) Normalized Raman (d) and SERS (e) spectra of cystine (disulfide stretching mode region, alkaline solutions). Relative Gaussian areas for the six different S–S bond conformations are indicated in brackets. Adapted from [36]. Copyright 2014 Springer.

An intriguing aspect in the work of Grass and Treuel [36] concerns the behavior of disulfide bonds on the surfaces of their NPs. By using the ratio of C–S to S–S bond signals as an indicator, they found that cysteine and its disulfide-linked dimer cystine (Figure 2a) produce the same signal in the surface bound state. As expected, this situation was found to depend on the presence of oxygen but was completely independent from the starting conditions, i.e., from adding the dimer or the monomer to the suspension. Taking a closer look at the C–S–S–C bond rotamers and comparing the Raman signal of the free molecule in solution (compare Figure 2), to the SERS signal of the surface bound molecules, they found that only few dominant rotamers were present in the surface bound molecules while the free molecules showed a Boltzmann type distribution.

Remarkably, they found a different behavior for the S–S bond motifs present in the insulin molecule. The molecule contains three S–S bonds in different conformations and the Raman spectra (Figure 2b) show that they are indeed contributing almost equally to the overall protein signal in this part of the spectrum. The adsorption process changes this situation significantly (Figure 2c) and only two remaining conformations contribute significantly to the overall spectrum of the surface-bound insulin.

Their data further revealed the involvement of individual functional groups and, specifically, the carboxyl group in the interaction process. They also provided a detailed evaluation of the amide- and C–C bands, linked to a loss of secondary structure, corresponding well to the findings reported using other, less surface sensitive, techniques [6,47,51,53–59].

From their collective data, Grass and Treuel could provide convincing clues, suggesting that the structural network of the protein prevents a more complete relaxation of the individual S–S bonds. Demonstrating the changes in secondary structure in the immediate vicinity of the surface, they provided, together with previous literature findings, strong indications that structural stress within the molecule indeed changes the behavior of the individual bonds. The simultaneous binding of many different functional groups that was found in their data, results in high avidity of the protein for the NP surface in addition to individual affinities of functional groups. The presumably reduced solubility of the desorbing protein further decreases its tendency to desorb. In this context, further experiments are desirable, dissecting the influences of avidity, affinity, van-der-Waals-type and Coulomb-type interactions.

### Role of surface chemistry

A plethora of different surface functionalities exists and structure–function relationships have yet to be established. Therefore, the modification of NP surfaces for the directed transport of drugs or vaccines has received much attention [71,128–133], but such modifications were largely performed without any detailed mechanistic knowledge regarding the bio-response.

Many studies focused on polymeric materials of synthetic [134–135] or natural origin [132,136–141]. An interesting result of these studies was that poly(ethylene glycol) (PEG) coatings reportedly increased the blood circulation time of intravenously administered NPs [142]. This finding was attributed to an exacerbated macrophage response as a consequence of reduced protein binding onto the PEG-coated surfaces [142]. Interestingly, the systemic behavior of PEG-coated NPs was also reported to be changed, demonstrated by a decreased renal clearance of PEG coated NPs [143], especially when long (>20 kDa) PEG chains were used [144]. We emphasize in this context that little is known about the exact physical and chemical properties of the PEG surfaces under such conditions, or about the consequences for their molecular scale interactions with proteins. This topic clearly deserves further attention.

Treuel and coworkers employed circular dichroism (CD) spectroscopy to study the thermodynamic and structural aspects of NP–protein interactions [47]. They investigated the formation of a serum albumin, an established model protein [7–9,80,111], corona around Au (13 ± 2 nm) and Ag (diameter 40 ± 10 nm) NPs, specifically addressing the effect of a (poly)vinylpyrrolidone (PVP) coating around the metallic surface on the protein adsorption/desorption equilibrium.

For citrate-stabilized Au and Ag NPs, they found affinities in a low nanomolar concentration regime (*K*_D(Au)_ = 33 ± 3.2 nM, *K*_D(Ag)_ = 20 ± 1.1 nM) [47], confirming an extremely high affinity of proteins towards these surfaces. When the PVP coatings were applied around the NPs, the affinities were found to be significantly lower (*K*_D(Au–PVP)_ = 0.5 ± 0.05 µM, *K*_D(Ag–PVP)_ = 0.2 ± 0.05 µM) [47].

These findings underline the importance of the original surface functionality of the NPs for their interaction with proteins. In addition, a further aspect emerges from the implications of these results: Only a stable and persistent molecular NP surface functionality can play a significant role in shaping the in vivo protein corona. While polymer coatings are regarded as relatively stable under such conditions, other ligands can well be replaced in equilibrium-type reactions, even under chemically less complex conditions [13].

It has been discussed before, how the formation of a protein corona can change the surface functionality of NPs and that these changes can affect NP translocation in biological systems [145–146]. Links between specific surface coatings and cytotoxic effects were empirically established [143,147–152] but the chemical composition of the NP core is also a decisive parameter. For silver NPs it was shown that their cytotoxicity is predominantly caused by the release of silver ions even when polymer coatings were applied [25,113,153]. The somewhat independent modes of action of the NP surface and the core need therefore to be considered in detail to assess the biological impact of NPs, an effect that is still often overlooked.

### Role of Coulomb interactions in corona formation

The role of Coulomb-type forces for the interaction process between NPs and proteins has been investigated and the contribution of overall protein charge for this behavior has been discussed [28,154–157]. Recently, even the role of net protein charge for the composition of the serum corona around negatively charged silica NPs has been addressed in a comprehensive study, however, without finding enhanced binding of proteins that were overall positively charged at pH 7.3 [8].

Moreover, it was suggested that charge distributions on the protein surface, rather than the overall molecular charge, govern the Coulomb interactions between NP and protein [9]. This intriguing idea seems indeed very relevant, considering that the Debye length at the typical ionic strengths of biological fluids and PBS buffer is below 1 nm, thus, smaller than the typical size of a protein (few nanometers).

Further refining this idea in a different experimental approach, Treuel et al. chemically altered the surface charge distribution of HSA and studied the effect on the corona formation around dihydrolipoic acid (DHLA)-coated quantum dots (QDs) by using fluorescence correlation spectroscopy (FCS) [4].

**Figure 3:**
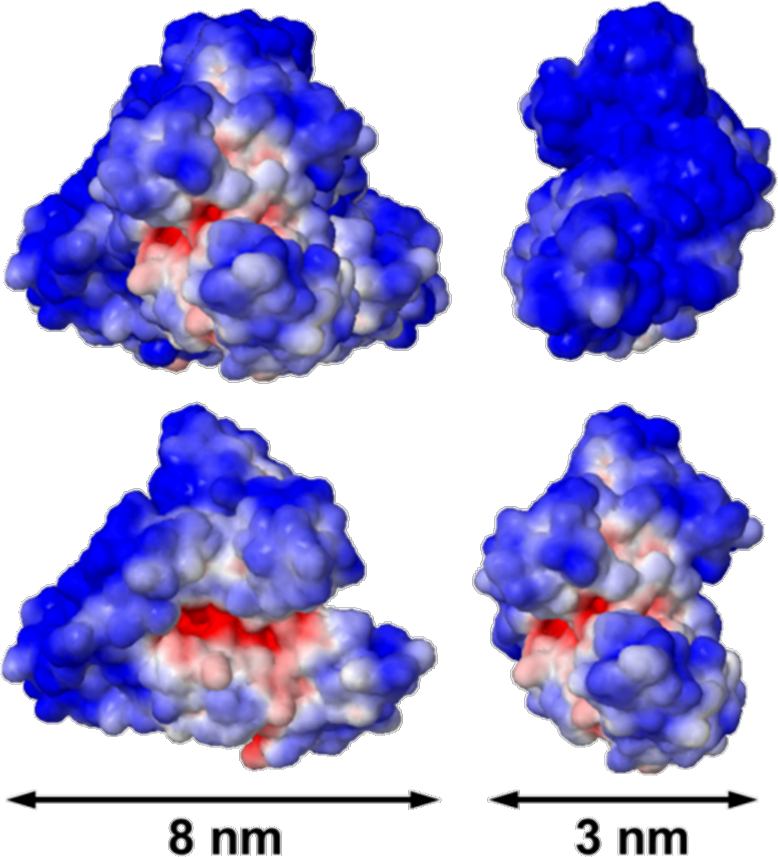
Human serum albumin (HSA, PDB code: 1UOR) represented as space-filling models, colored to indicate surface electrostatics at pH 7.4 (blue, negative potential; red, positive potential; range from −5 *k*_B_*T*/*e* to +5 *k*_B_*T*/*e* (calculated online at http://nbcr-222.ucsd.edu/pdb2pqr/49 [158–159]). Reprinted with permission from [4]. Copyright 2014 American Chemical Society.

The electrostatic surface potential of native HSA shows distinct, positively charged patches on an otherwise negative potential surface (Figure 3). These patches are caused by the presence of basic lysine and arginine residues carrying a positive charge in their side chain at physiological pH. Negative surface potentials arise from aspartic- and glutamic-acid residues carrying a dissociated carboxyl function and, hence, a negative charge, in their side chain under physiological conditions.

By reacting the ε-amino group of lysine with succinic anhydride, Treuel et al. [4], turned these positively charged groups into negatively charged carboxylate functions, obtaining succinylated HSA (HSAsuc). In addition to the surface charge distribution, this succinylation changed the overall zeta potential of the HSA molecule from (−10.5 ± 1.3) mV for native HSA to (−19 ± 4) mV for HSAsuc (both in PBS at pH 7.4).

For comparison, they also altered the carboxyl groups of the native HSA molecule by reacting them with ethylenediamine, thus, converting them into positively charged amino groups, creating an aminated HSA molecule (HSAam). This amination expectedly decreased the magnitude of the zeta potential of native HSA, (−10.5 ± 1.3) mV, to (−6.1 ± 0.4) mV (in PBS at pH 7.4). DLS measurements confirmed that the protein size remained essentially unchanged after all chemical modifications and the overall protein fold was preserved.

**Figure 4:**
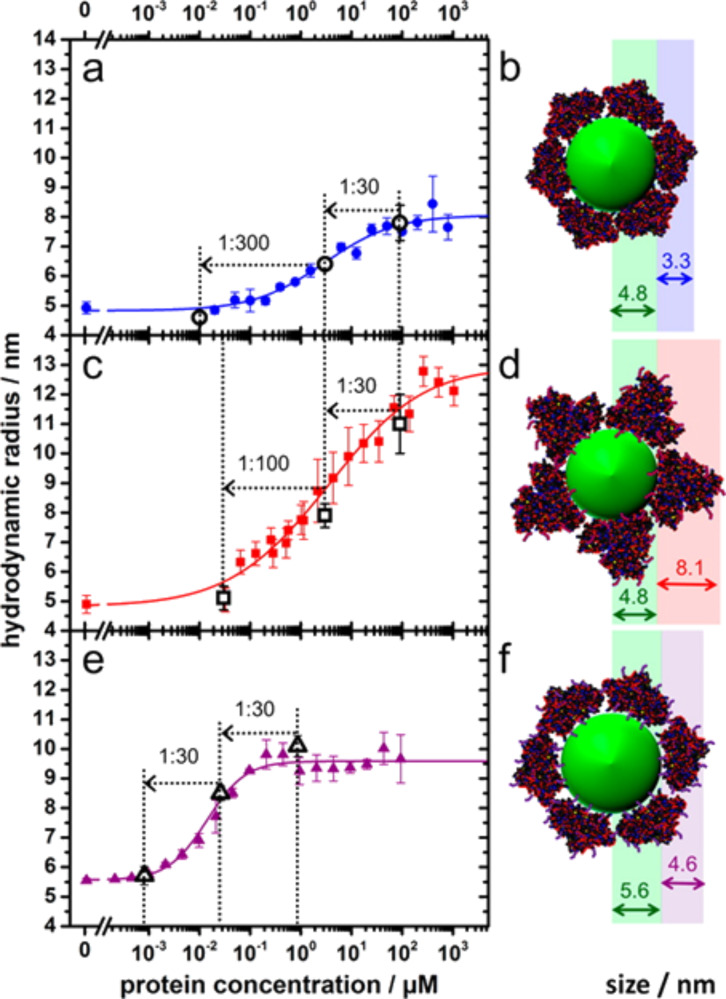
Binding curves as determined by fluorescence correlation spectroscopy and schematic representations for adsorption of (a and b) HSA, (c and d) HSAsuc and (e and f) HSAam onto dihydrolipoic acid-coated quantum dots. Filled symbols: Hydrodynamic radii of DHLA-QDs plotted as a function of the concentration of (a) HSA, (c) HSAsuc and (e) HSAam free in solution. Solid lines represent fits of the modified Hill equation to the data [4]. Open symbols: Hydrodynamic radii measured at 90 μM (a) HSA and (b) HSAsuc and 0.9 μM (e) HSAam and after two successive dilution steps. (b, d, and f) Schematic depictions of the hydrodynamic radii increase as a result of protein adsorption, with (b) native HSA and (f) HSAam (adsorbing with their triangular faces, leading to a radius increase of 3.3 and 4.6 nm, respectively), and (d) HSAsuc adsorbing with the edge of the prism (causing a radius increase of 8.1 nm). Reprinted with permission from [4]. Copyright 2014 American Chemical Society.

They then used fluorescence correlation spectroscopy to measure binding curves for the adsorption of native and modified HSA to DHLA-coated QDs. Intriguingly, these relatively simple chemical modifications of the proteins surface charge distribution, were found to dramatically change the nature of protein adsorption onto their NPs (Figure 4).

The adsorption of native HSA led to a radius increase due to protein adsorption of Δ_rh_ = (3.3 ± 0.6) nm, commensurate with a complete surface coverage by HSA molecules adsorbing with their triangular faces to the QDs and well in line with similar experiments [9,111]. From the quantitative analysis of their data with the Hill equation [111], they revealed an equilibrium constant of *K*_D(HSA)_ = (6.4 ± 3.6) μM, supporting previous findings for HSA adsorption onto polymer-coated FePt NPs [9,111]. The data from HSAsuc showed a distinctly different behavior: the radius increase due to HSAsuc adsorption was found to be Δ_rh_ = (8.1 ± 0.6) nm, which could be explained by binding of HSAsuc molecules to the QD surface in a “side-on” orientation with the triangular sides perpendicular to the surface. The corresponding equilibrium constant was quantified to *K*_D(HSAsuc)_ = (19 ± 8) μM.

For the aminated HSA, HSAam, they found even more drastic deviations from the behavior of the native molecule. The change in radius was quantified to Δ_rh_ = (4.6 ± 0.1) nm, thus, larger than for native HSA but smaller than for HSAsuc. Since additional amine functions in the protein are expected to randomly enhance already present positive patches or generate entirely new ones, a variety of HSAam orientations results thus, explaining the increased corona thickness. Remarkably, the equilibrium constant for the adsorption of HSAam to the QD surfaces was found to be *K*_D(HSAam)_ = (22 ± 3) nM commensurate with a 1000-fold increase in binding affinity compared to the native HSA. This is particularly striking, considering that the net charge of the protein remains negative even after amination, and was explained by a higher density of positive charges on the protein surface.

The confirmation of the notion that charged patches, rather than overall protein charge, govern the binding efficiency between NP and protein, but also the intriguing demonstration how this behavior can be changed by chemical modifications, pave the way for a more structure–function-based interpretation of corona formation.

In addition Treuel, Nienhaus et al. addressed a further point, critically important to our description of the corona formation: reversibility of protein adsorption [4]. By diluting their samples in several steps after the full corona was formed (see Figure 4a,c,e) and measuring the resulting corona thickness after an equilibration time (90 min), they were able to demonstrate the full reversibility of the protein adsorption for native HSA and both chemically modified HSA versions. This is the first quantitative data, convincingly demonstrating that protein adsorption onto NP surfaces may indeed be validly treated as an equilibrium process. A word of caution is, however, in order here: Depending on the NP surface type, denaturation of the protein may occur [7,36,47,81] and, depending on the degree of this denaturation, could cause severe deviations from a true equilibrium behavior. It is noteworthy in this context that even for the aminated HSA with an equilibrium constant in the range of nanomoles, indicating an extremely high affinity of the protein towards the QD surface, Treuel, Nienhaus and co-workers were able to demonstrate full reversibility. The fact that this is even true for such high affinity interactions is a further indication that denaturation of proteins may be a centrally decisive factor for any non-equilibrium effects observed in corona formation [10].

### Particle size determines corona composition

**Figure 5:**
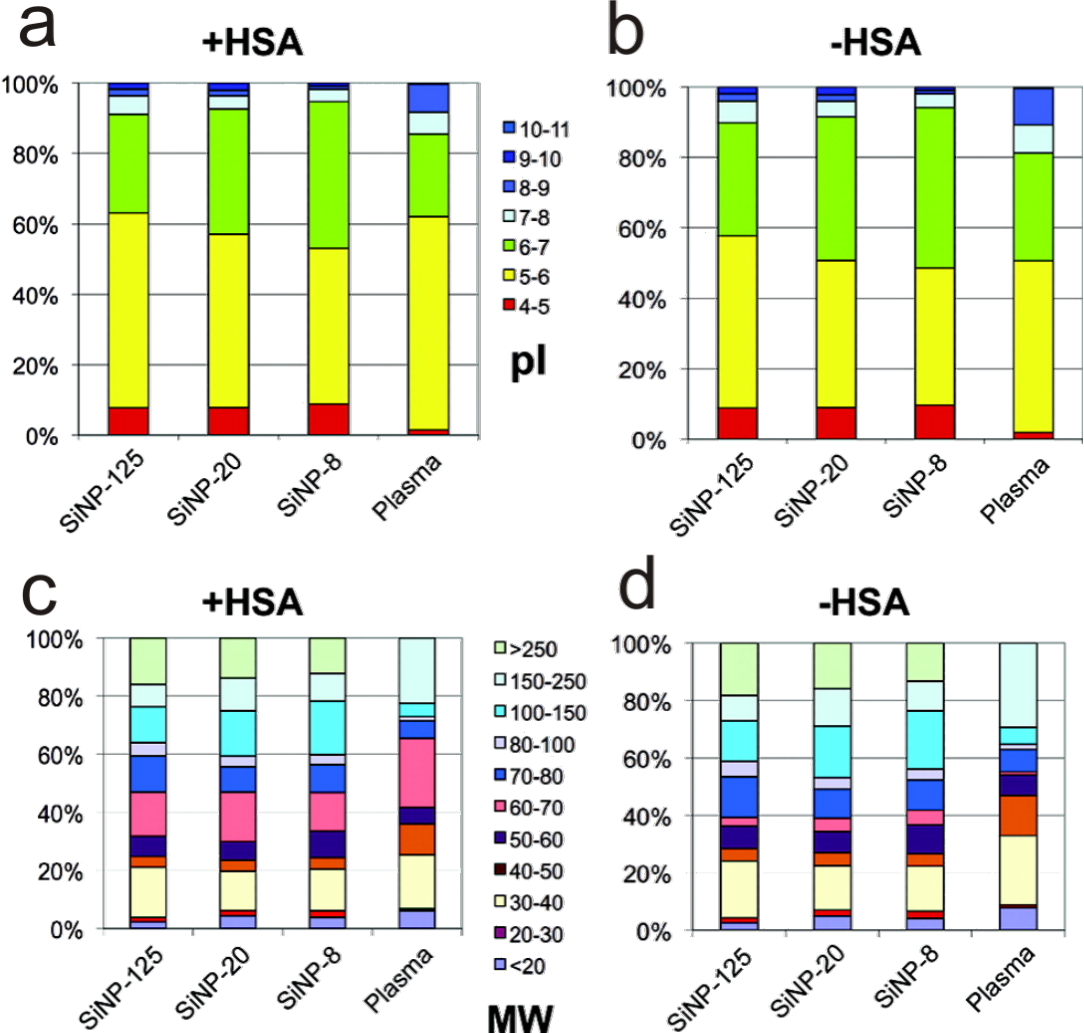
Composition of protein coronae around SiNPs of different sizes as identified by quantitative mass spectrometry by Tenzer et al. [8]. Proteins were classified by (a,b) their isoelectric points (pI) as calculated from the sequence information and by molecular weight (MW) (c,d). (a,b): SiNPs preferentially bound negatively charged proteins (pI < 7) at pH 7.3. Proteins with pI < 5 were enhanced in the protein corona, independent of the particle size, as compared with plasma. (c,d) Proteins with low molecular mass were found to be less abundant in the corona than in plasma, while large proteins were significantly enriched in the corona. The observed patterns remained unchanged when the most abundant plasma protein, human serum albumin (b,d: −HSA), was excluded from the data analysis. Reprinted with permission from [8]. Copyright 2014 American Chemical Society.

For a deeper understanding of protein corona formation under physiological conditions it is essential to identify the underlying individual factors governing the corona under these conditions. In 2011, Tenzer and colleagues discovered that the size of the particles alone is a critical physicochemical determinant of the human blood plasma corona [8]. They could show that even differences in silica nanoparticle (SiNP) size of only 10 nm significantly affected the protein corona composition around three different SiNPs (with diameters of about 20, 30, and 110 nm). One strategy to interpret their results was to classify the SiNP-specific protein signatures, as identified by quantitative mass spectrometry, by their calculated isoelectric point (Figure 5).

At the physiological pH of blood plasma (pH 7.3), preferential binding of negatively charged proteins (pI < 7) onto the SiNP surfaces was reported. The protein size also played a significant role: While proteins with a high molecular mass were enriched on the NP surfaces as compared to their abundance in the plasma, proteins with low molecular mass were less abundant in the corona than in the surrounding plasma (Figure 5c,d).

Of all identified corona proteins 37% showed significant differences in their binding behavior towards the NP surface as the NP size was altered. The proteins that show such size dependence in their adsorption behavior are involved in all kinds of biological processes, hence, they could not be attributed to a functional class. Interestingly, they were able to identify individual proteins (e.g., prothrombin or gelsolin) showing a higher affinity for larger SiNPs (110 nm) but also other proteins (e.g., clusterin) with higher affinities to smaller SiNPs (20 nm).

Many other proteins such as immunoglobulin (Ig) G or actin showed no dependence on the NP size. The complexity of protein corona formation under physiological conditions is impressively emphasized by this study: A single physical parameter such as the NP size can dramatically change the composition of the protein corona around NPs that are otherwise identical. Intriguingly, the detailed evaluation of the data did not confirm the previously suggested [22,43,160–161] trivial link between the binding profiles on the NPs and the relative protein concentrations in the plasma.

### The dynamics of corona formation and composition

The physicochemical properties of the protein corona and its evolution with time have been shown to affect the behavior of nanoparticles in biological environments to a large extent [1,11,28,162]. Our current understanding suggests that initially a “soft” protein corona, with a highly dynamic exchange of proteins, is formed around NPs. Changing its composition over time, the “soft” corona slowly evolves to a “hard” corona with less dynamic exchange [112,162–163]. Previous studies investigating nanomaterial exposure to complex biological environments [3,8,41,112,162] have largely failed to allow for the highly dynamic situation of physiological systems. Corona-carrying NPs may need to instantly react to external stimuli under such conditions as exemplified by considering the blood system where processes controlling haemostasis and thrombosis may be triggered on a timescale of minutes [28].

Tenzer and co-workers [10] recently developed a new experimental approach allowing for a time-resolved determination of NP-specific fingerprints. This method, based on label-free liquid chromatography mass spectrometry (LC-MS), allows for an insight into the time-evolution of the full serum protein corona. Such information, especially for short exposure times, is urgently needed to understand the interactions between NPs and biological systems. Briefly, they used their LC-MS based method to measure time-resolved corona compositions on silica and polystyrene NPs in human serum. By using NPs of various sizes (diameters of about 35, 120, 140 nm), and surface functionalities (amine, carboxylate, unmodified), they were able to provide insights into the consequences of these factors for corona composition and dynamics.

They demonstrated in their study that coronas were formed very rapidly, and showed an unexpected complexity regarding composition and dynamics on all of the NPs under investigation. They quantitatively detected 166 different plasma proteins within the corona after 0.5 min. The number of individual proteins quantified in the corona increased to almost 300 on the different types of NPs at prolonged exposure times (up to 480 min).

**Figure 6:**
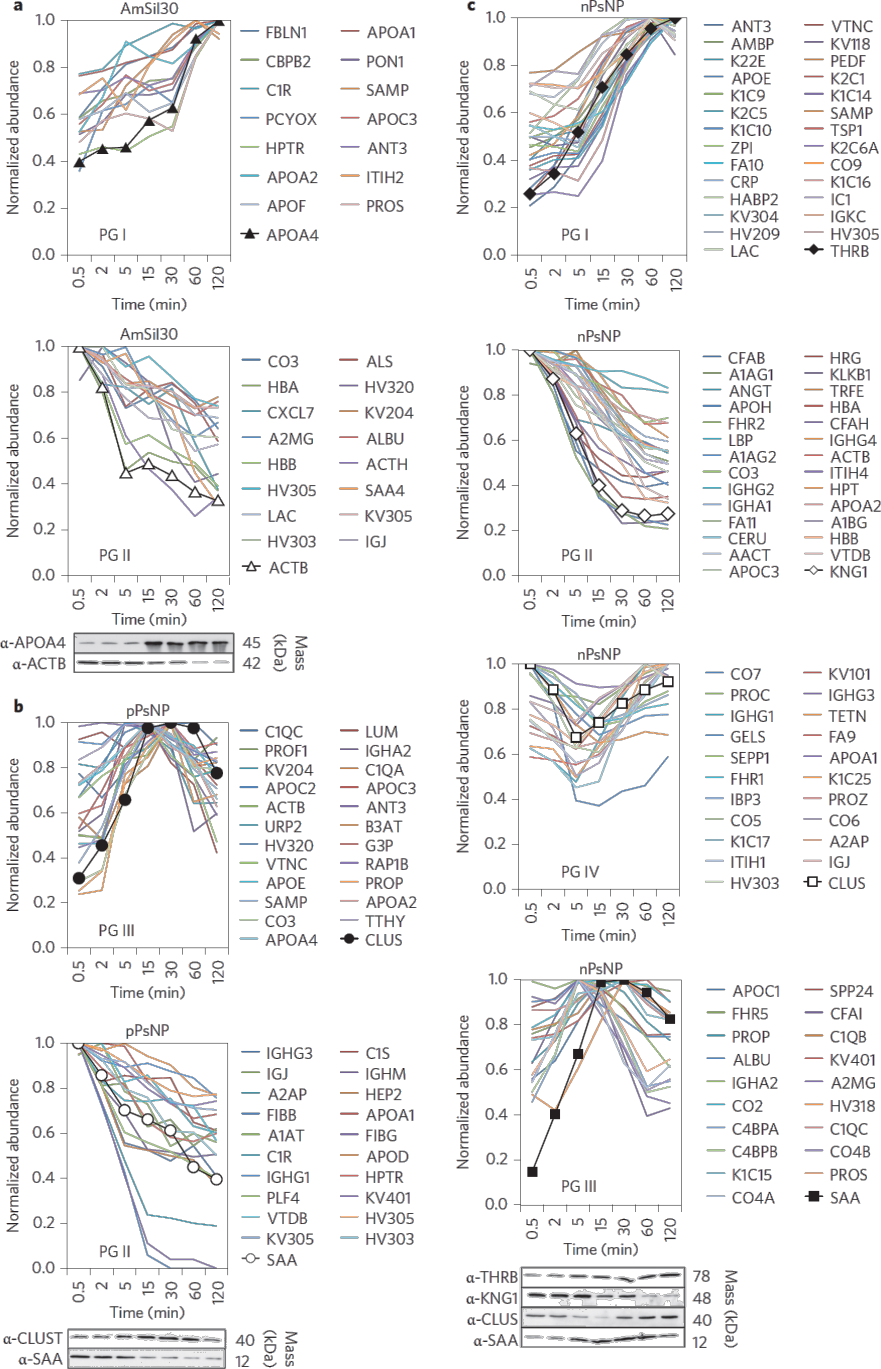
Tenzer et al. [10] revealed in a correlation analysis distinct kinetic protein-binding modalities during the temporal evolution of the serum protein corona. (a–c): Protein abundance profiles of AmSil30 (SiNP), time smoothes and normalized signals. (a): pPsNPs (polystyrene NP) and (b): nPsNPs (polystyrene NP) (c): NP coronae were classified into four groups by correlation analysis and relative values were normalized to the maximum amount across all time points for each protein. Protein groups PG I and PG II showed increasing or decreasing binding over time, respectively. PG III, ‘Peak’ proteins, display low abundance at the beginning of plasma exposure and at later time points, but higher (peak) abundance at intermediate time points. PG IV proteins show the opposite behavior, with a high abundance at early and late time points, but low abundance at intermediate time points. Profiles were verified independently for representative candidates (marked by symbols) by immunoblot analysis. Reproduced with permission from [10]. Copyright 2013 Nature Publishing Group.

The previous understanding of such compositional changes has been shaped by Vroman in 1962 [164] who described the time-dependent composition of a protein layer. This description is commonly referred to as “Vroman effect” and it suggests that the bound protein layer is dominated by highly abundant proteins at short exposure times which are later replaced by less abundant proteins with a higher affinity to the surface [165]. The resulting dynamic exchange between bound and unbound predicted by this model should, in this notion, be controlled by the association and dissociation constants of various individual proteins. As a consequence of this behavior, the abundance of certain individual proteins within the protein surface layer would increase or decrease with time. The results of Tenzer and colleagues confirm this effect by identifying protein groups that indeed show increased or reduced binding with an increasing exposure time (see Figure 6).

However, not all binding kinetics found in their study seemed to be solely explained by the Vroman effect: Individual proteins showed a different behavior with a low abundance at short exposure times, a peak “abundance” at intermediate times and an again decrease abundance at long exposure times (Figure 6). Other proteins were also observed to be highly abundant at short and long exposure times with a minimal abundance at intermediate exposure times.

A further intriguing aspect of their results was the finding that the composition of the corona only changed in a quantitative matter rather than in a qualitative fashion. From the complexity of their findings, also with regard to effects of surface composition and size, it seems that the interplay of different physicochemical parameters rather than individual factors govern the overall situation. Since, in addition to the these factors, protein exposure time was confirmed as a critical factor, a multi parameter classifier will likely be required to model NP–protein interaction profiles in biological relevant environments [10,162].

A direct link between these findings and their (patho)biological relevance was also established in this study by in vitro experiments by using primary human cell models of the blood system. Pristine NPs immediately affected the vitality of endothelial cells, triggered thrombocyte activation and aggregation, and resulted in hemolysis of erythrocytes. However, such pristine NPs existed only for a very brief period of time in the biological environment. The formation of the biomolecular corona around the NPs modulated their behavior: cells of the blood system seemed to be protected against NP-induced (patho)biological processes by the presence of the corona and also cellular uptake could be promoted [10].

### Effect of the corona presence and composition on cellular uptake

The effect of protein corona formation on the cellular uptake of NPs has been in detail addressed by numerous studies [2,5,7,10,12,166–169]. The current situation, largely relying on in vitro findings, indicates that the presence of the corona indeed alters the cellular response to NPs. For example, NP opsonization with immunoglobulin reportedly promoted receptor-mediated phagocytosis by macrophages [155]. The presence of polyethylene glycol (PEG) was shown to suppress protein absorption resulting in a decreased uptake by macrophages [142] and longer circulation times in the blood as well as altered bio-distribution upon injection in mice [155].

Attempts were also made to coat NPs by specific proteins. For example, transferrin, well known to be internalized via its cognate receptor, was used to create a protein corona and study its effect on cellular uptake [11–12]. To elucidate how the presence of a protein adsorption layer around NPs affects their cellular uptake, Jiang, Nienhaus et al. [12] compared the uptake of small (diameter about 10 nm), carboxyl-functionalized polymer-coated FePt NPs (fluorescently labeled by DY-636 dye molecules in the polymer shell), by live HeLa cells in the presence or absence of human transferrin (TF) and human serum albumin (HSA) in phosphate-buffered saline (PBS) medium. They studied the uptake of the NPs by quantitative confocal fluorescence microscopy. For comparison, they also studied the cellular uptake of fluorescently labeled (ca. 1:1 ratio) transferrin and HSA molecules. Whilst transferrin was endocytosed in significant amounts, HSA was barely internalized by HeLa cells under otherwise identical conditions. In contrast, the uncoated NPs were taken up in large amounts, whereas the presence of an HSA or transferrin corona both reduced the amount of endocytosed NPs with the exact causes for this behavior remaining uncertain.

Also focusing on the transferrin corona, Salvati, Dawson and co-workers were able to demonstrate how transferrin-functionalized NPs can lose their targeting capabilities when a biomolecule corona adsorbs on their surface [11]. They studied the uptake of transferrin-decorated SiNPs with PEG8 spacers by A549 lung epithelial cells. After adding bovine serum to their experiments, proteins from the surrounding medium reportedly formed a corona around their pre-functionalized NPs shielding transferrin from binding to both its cognate receptors on cells and also to soluble transferrin receptors. While NPs were still taken up by the cells, the targeting specificity of transferrin was lost. These findings underline very well, the complexity of the situation where even protein mediated cell-targeting suffers from corona formation under physiological conditions. Future approaches need to work around these effects and a detailed mechanistic knowledge is needed in order to do so.

**Figure 7:**
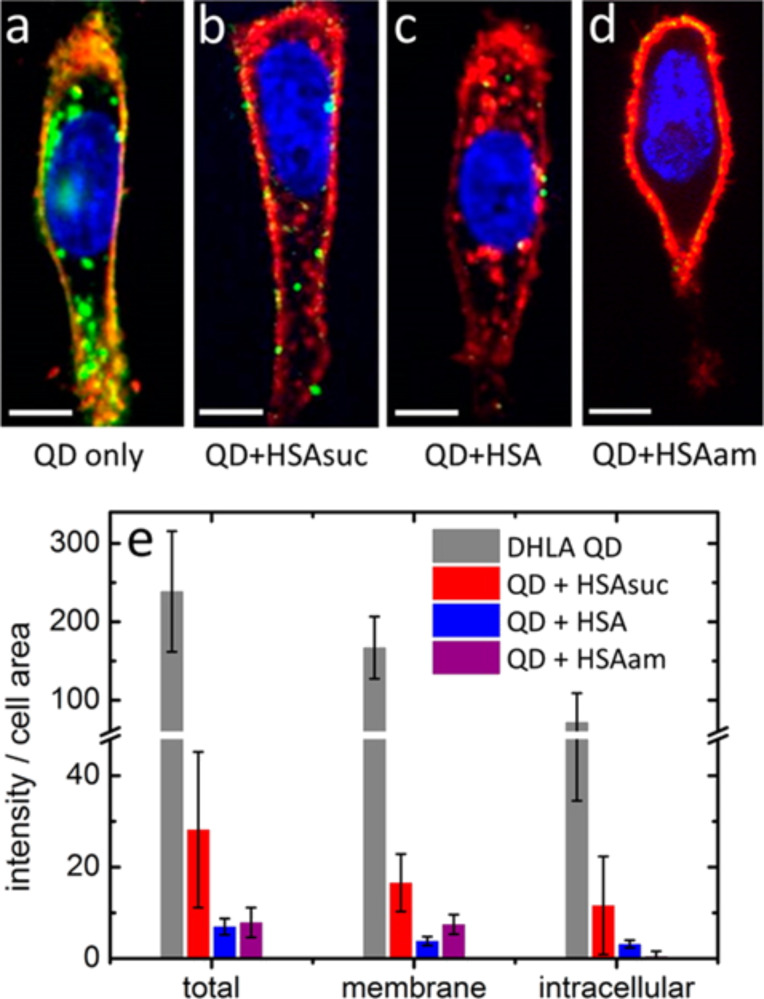
Fluorescence microscopy images (a–d) of the cellular uptake of DHLA-QDs by HeLa cells. Cells were incubated for 2 h with 10 nM QDs in PBS (a) without protein, (b) with 100 μM HSAsuc, (c) with 100 μM native HSA and (d) with 100 μM HSAam (red: cell membrane blue: nucleus, green: QDs). Scale bar: 10 μm. (e) Quantified evaluation of NP uptake after 2 h. Error bars represent standard deviations due to variations between individual cells. Reprinted with permission from [4]. Copyright 2014 American Chemical Society.

In a different approach, Treuel, Nienhaus and co-workers [4] studied the uptake of DHLA coated QDs by HeLa cells, comparing the uptake of the as-synthesized NPs to the cellular uptake of the same NPs carrying an HSA corona or a corona consisting of aminated (HSAam) or succinylated (HSAsuc) HSA, respectively, as described above. The cellular uptake was studied by confocal fluorescence microscopy and fluorescence intensities were quantified for the membrane associated and intracellular fractions of QDs. Representative cell-images are shown in Figure 7.

Their results revealed that the unfunctionalized NPs were taken up in the largest amounts while the presence of all coronae reduced the cellular uptake. A possible contribution to this observation is that, in presence of free proteins, the cellular endocytosis machinery was also occupied with internalization of the freely dissolved protein. However, intriguing differences were found between the effects of the different coronae, essentially all consisting of HSA with just minor chemical modifications as described above.

Internalization of HSAam-coated NPs by the cells was completely suppressed within the sensitivity limits of their experiment. In addition the time-dependent NP uptake by HeLa cells was investigated, using spinning disk confocal microscopy. In these experiments, all QDs were found to accumulate at the cell membrane within minutes after exposure. However, the kinetic analysis of this process showed characteristic times for QD association to the membrane to differ by more than one order of magnitude. Rate coefficients were also determined for internalization of NPs and varied less than a factor of 2. The combined interpretation of their data, allowed them to deduce that the overall uptake is controlled by the binding of the NP to the cell membrane.

These findings will clearly help to design NPs for directed cellular uptake. The notion that rather the kinetics of membrane binding can be affected by the presence and nature of the protein corona, than the kinetics of the actual endocytosis process, allows novel strategies in this context. The role of this finding for the passive uptake of NPs by cells, remains unresolved, however, NP-membrane interactions will likely also play a central role here.

## Conclusion

Significant progress has been made towards understanding the protein corona formation around NPs, yet, the current state of knowledge clearly emphasizes the continuous need for further molecular-scale studies of protein adsorption to engineered NPs.

The effect of protein corona formation on colloidal stability has been investigated and remains a very relevant problem for any studies under physiological conditions. While we have discussed first mechanistic insights in this review, further work along these lines is needed in order to understand molecular mechanisms and to identify the NP-related parameters governing this behavior. Not only the efficiency of the corona formation but possible individual proteins contribution to inter-particle-bridging and denaturation of proteins may be decisive factors in this respect. We have discussed recent advances showing that protein adsorption can indeed be validly described as an equilibrium process but we point out that this may strongly depend on the properties of the NP surface and also on the individual properties of different proteins. It remains a focus of current research in this context to identify the possible parameters, conditions and forces that may cause deviations from a pure equilibrium or lead to kinetic hindrance on specific NP surfaces.

Size alone and, hence, surface curvature has been identified as one factor governing the composition of the protein corona under physiological conditions [8]. While the mechanistic aspects of this finding remain unclear on a molecular scale, the biological implications may be dramatic. As discussed here, recent findings show that the interaction between corona carrying NPs and the cellular plasma membrane plays a central role for cellular uptake [4]. Moreover, it has been suggested that smaller NPs need to exceed a critical threshold concentration on the plasma membrane before the internalization process is triggered [170]. Understanding the role of the physiological protein corona and its NP-size dependent composition will clearly be an important aspect of future work in this area.

The individual contribution of functional groups within proteins has received some attention and a central aspect along these lines will be our understanding of the relation between the affinity of individual groups to the NP surface and the affinity of the protein, which is additionally governed by avidity effects. This remains a formidable task, since so many factors, including protein unfolding, entropic effects and effects related to the competitiveness of protein adsorption add further complexity to this issue under physiological conditions.

First relations between NP and protein properties and the resulting corona have been established amongst which the effect of Coulomb interactions between NP surfaces and charged patches on protein surfaces deserve special attention. The influence of this factor on the proteins affinity to the NP surface but also on the nature of the resulting corona and on cellular uptake has been demonstrated and the extent of this effect is indeed striking. A major challenge in this respect is the link between the results from the idealized conditions of biophysical experiments and the full complexity of the physiological situation. To identify and understand the individual factors governing this behavior is therefore an important task for future research in this field. Understanding the delicate interplay of these individual contributions in shaping the physiological situation is of paramount importance, yet, the long and continuing struggle of statistical mechanics in fully explaining the ensemble observations of thermodynamics serves as an impressive reminder of the complexity that can arise from attempting such seemingly trivial links.

The fact that the serum corona shows the complex temporal effects discussed above which cannot fully be explained on a molecular scale further underscores the complexity of the topic.

Understanding the formation and composition of the corona remains a challenging task but also the biological consequences of the corona and its composition need to be revealed in detail. Establishing structure function-relationships linking NP properties to biological response therefore remains a still distant goal despite the considerable achievements of the past years. This knowledge is needed not only to understand and minimize nano-toxicity but also to develop NPs for desired transport in nano-medical applications.
